# Intussusception secondary to Peutz-Jeghers syndrome: a case report and literature review of diagnostic and therapeutic advances

**DOI:** 10.3389/fmed.2025.1687958

**Published:** 2025-10-21

**Authors:** Jing Zhang, Tian-Hao Xie, Yan Fu, Xiao-Shi Jin, Qiang Wang, Zheng Niu

**Affiliations:** ^1^Department of General Surgery, Affiliated Hospital of Hebei University, Baoding, Hebei, China; ^2^Basic Research Key Laboratory of General Surgery for Digital Medicine, Affiliated Hospital of Hebei University, Baoding, Hebei, China; ^3^Department of Ophthalmology, Baoding No.1 Central Hospital, Baoding, Hebei, China

**Keywords:** Peutz-Jeghers syndrome, gastrointestinal intussusception, surgical polypectomy, general surgery, case report

## Abstract

Peutz-Jeghers syndrome (PJS) is a rare autosomal dominant genetic disorder characterized by gastrointestinal hamartomatous polyposis and mucocutaneous pigmentation, predisposing patients to malignancies. This article reports a case of a 19-year-old male presenting with recurrent abdominal pain, diagnosed with small intestinal intussusception secondary to PJS polyps. Computed tomography revealed the “double-ring sign,” indicative of intussusception, and laparoscopic exploration confirmed multiple small intestinal polyps, which were subsequently resected. Postoperative pathology confirmed the diagnosis of PJS. The article reviews the diagnostic criteria, genetic basis, clinical manifestations, and recent advances in the management of PJS. It highlights the transformation in management strategies, driven by advancements in genetic testing, endoscopic interventions, and multidisciplinary collaboration. Early diagnosis, genetic screening for first-degree relatives, and lifelong surveillance are emphasized to mitigate the risk of malignancies and improve patient outcomes. The refinement of diagnostic criteria, genotype–phenotype correlations, and innovations in endoscopic techniques are discussed, underscoring their role in optimizing therapeutic approaches.

## 1 Introduction

In 1921, Peutz first identified gastrointestinal polyps and skin pigmented spots in three generations of a Dutch family (1). In 1949, research by Jegher confirmed that this rare disease was an autosomal dominant genetic disorder conforming to Mendelian inheritance laws (2). In 1954, the disease was officially designated as Peutz-Jeghers syndrome (PJS) (3). The estimated incidence of PJS ranges from 1 in 50,000 to 1 in 200,000 births, with no significant gender or racial differences (1). Skin pigmentation and multiple hamartomatous polyps in the gastrointestinal tract are the two most prominent clinical manifestations of PJS (4). Most cases result from mutations in the STK11 tumor suppressor gene (5). Over the past three decades, research on this rare disease has exhibited rapid and sustained growth (6).

A clinical diagnosis of PJS is established when one of the following criteria is met (4, 7): (1) two or more pathologically confirmed PJS polyps; (2) any PJS polyp in conjunction with a family history of PJS; (3) characteristic mucocutaneous pigmentation (involving the mouth, lips, nose, eyes, genitalia, or fingers) associated with a family history of PJS; or (4) any PJS polyp identified in a patient exhibiting characteristic mucocutaneous pigmentation. In addition to these clinical criteria, genetic testing, comprehensive clinical history review (including childhood records), and familial pedigree analysis constitute critical components of the diagnostic workup. Genetic screening is strongly recommended for first-degree relatives of individuals with confirmed PJS to facilitate early detection and intervention.

PJS polyposis predominantly affects the stomach (74.4%), small intestine (96.5%), and colorectum (78.6%), with rare occurrences in the gallbladder, nasal cavity, and uterus (8). Symptoms such as abdominal pain, intestinal obstruction, and gastrointestinal bleeding caused by polyps are common, with larger polyps correlating to earlier symptom onset. Surgical interventions are performed in 76.7% of cases between the ages of 6 and 25 years. The primary indications for initial surgery include intussusception (81.4%), obstruction (14.0%), and gastrointestinal hemorrhage (4.7%) (9). This article presents a case report detailing intussusception secondary to PJS polyps and offers a literature review of the latest research advancements regarding this condition, with the objective of enhancing clinical awareness and deepening understanding of the disease among healthcare professionals.

## 2 Case report

A 19-year-old male was admitted to the hospital due to “recurrent abdominal pain for 1 year with aggravation for 1 day.” Over the past year, the pain had been intermittent and dull, predominantly localized around the umbilicus, without radiation to other sites. The pain could resolve spontaneously. Both flatus and bowel movements were normal, with no pus or blood detected in the stool. Over the past year, the patient had not undergone any medical examinations or treatments. The day prior to admission, the abdominal pain worsened, prompting him to seek medical attention. The patient’s maternal grandfather had a history of rectal cancer. Apart from this family history, the patient had no known medical, surgical, psychosocial, or medication history.

**Figure 1:**
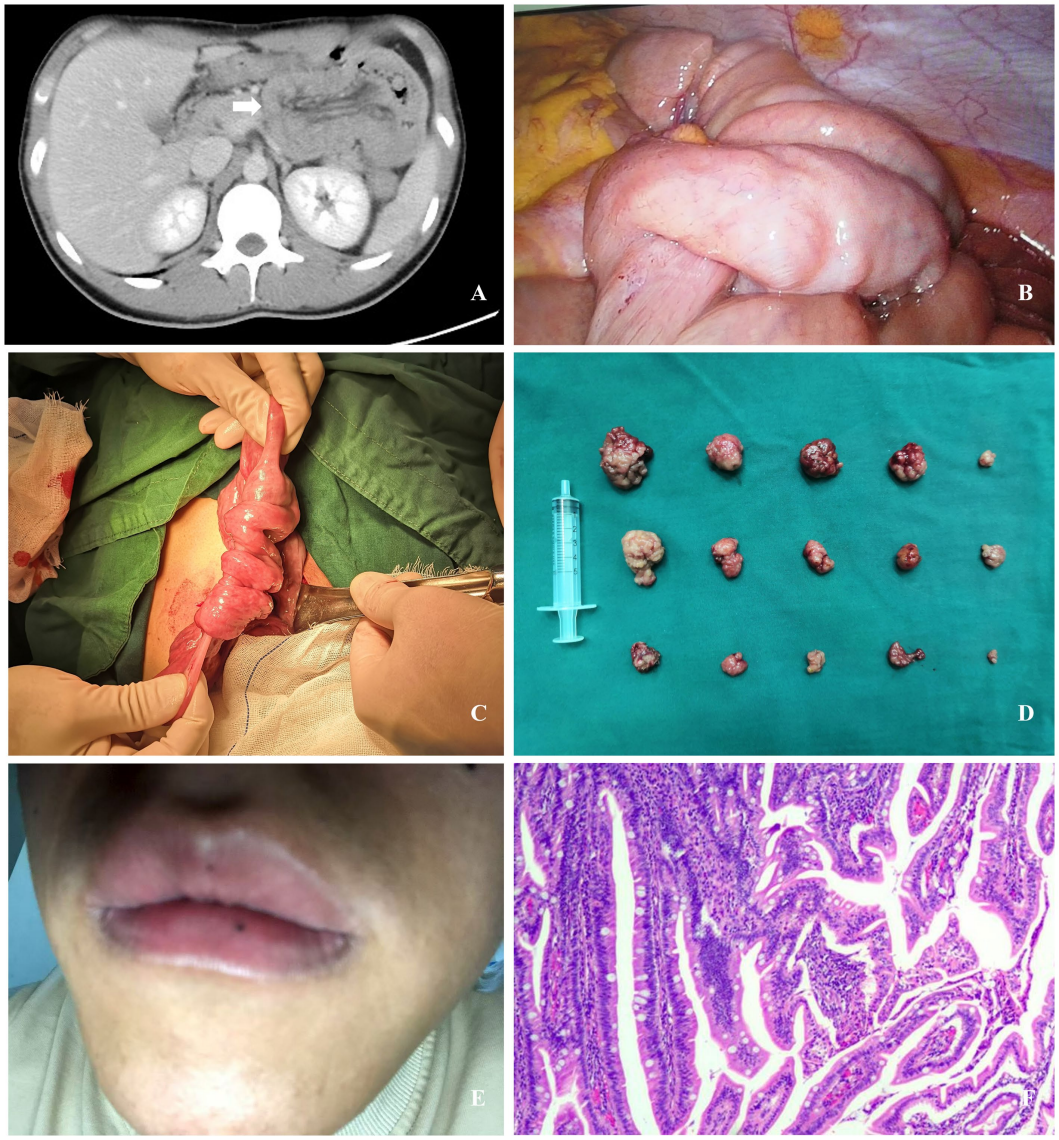
**(A)** Computed tomography revealed a “double-ring sign” in the intestinal loops within the left abdomen (white arrow). **(B)** Multiple instances of small intestinal intussusception were identified during laparoscopic exploration. **(C)** Small intestinal intussusception was identified during laparotomy. **(D)** Fifteen polyps were resected during the procedure. **(E)** A black macule measuring approximately 1.5 mm in diameter on the lower lip (the photograph was self-taken and provided by the patient post-discharge). **(F)** Postoperative pathology revealed hamartomatous polyps containing multiple small foci of low-grade intraepithelial neoplasia.

Laboratory investigations, including tumor marker profiling (alpha-fetoprotein, carcinoembryonic antigen, carbohydrate antigen 125 [CA125], CA15-3, CA19-9, and CA72-4), as well as routine hematology, liver function, and renal function tests, showed no abnormalities. Computed tomography (CT) revealed a “double-ring sign” in the intestinal loops at multiple levels within the left abdomen, indicative of intussusception (Figure 1A). Physical examination demonstrated a soft abdomen with mild tenderness localized to the left upper quadrant. No rebound tenderness or muscular rigidity was appreciated, and no palpable mass was identified. Digital rectal examination elicited no positive findings. During the hospitalization for observation, the abdominal pain partially resolved, and conservative management was initiated. On the third day of admission, the abdominal pain suddenly worsened. After obtaining informed consent from the patient, emergency surgery was performed.

Laparoscopic exploration was performed, revealing multiple small intestinal intussusceptions in the left mid-upper abdomen. The proximal small intestine appeared edematous, with dilated intestinal loops (Figure 1B). Following unsuccessful laparoscopic reduction of the intussuscepted small intestine, the procedure was converted to an open approach. Subsequent exploration confirmed no intestinal necrosis, thrombi, or mesenteric hematomas. Multiple intussusceptions of varying severity were identified in the small intestine at 15 cm, 50 cm, and 100 cm from the Treitz ligament (Figure 1C). After manual reduction and repositioning of the intussuscepted segments, further exploration revealed multiple small intestinal polyps. These polyps, ranging in diameter from 1.0 cm to 5.0 cm, were located at 15 cm, 25 cm, 35 cm, 100 cm, 120 cm, 140 cm, and 250 cm from the Treitz ligament. Small incisions were created in the intestinal wall at each polyp site, through which the polyps were extruded and then resected along with their stalks. A total of 15 polyps were excised (Figure 1D), and the intestinal wall defects were sutured with interrupted stitches.

Postoperatively, a whole-body physical examination revealed a black macule measuring approximately 1.5 mm in diameter on the lower lip (Figure 1E). The patient initiated oral water intake at 48 h postoperatively, was transitioned to a liquid diet on postoperative day 4, and advanced to a semi-liquid diet 1 week postoperatively. The postoperative course was uneventful, with no complications observed. The patient was discharged on postoperative day 10. Postoperative pathology (Figure 1F) revealed multiple hamartomatous polyps containing multiple small foci of low-grade intraepithelial neoplasia. No lesions were identified at the base of the stalks of pedunculated polyps. The final diagnosis was intussusception secondary to small intestinal polyps in the context of PJS.

During the 6-month follow-up period, video capsule endoscopy (VCE) confirmed the absence of polyps in the stomach, small intestine, or colon. The patient was explicitly advised on the necessity of lifelong surveillance, including specific protocols and intervals. The patient expressed satisfaction with the treatment outcomes and agreed to adhere to the recommended follow-up regimen.

## 3 Discussion

PJS is an autosomal dominant genetic disorder characterized by gastrointestinal hamartomatous polyposis, mucocutaneous pigmentation, and a high risk of malignancies across multiple organs. In recent years, its management strategies have undergone significant transformations driven by advancements in genetic testing technologies, optimization of endoscopic interventions, and the widespread adoption of multidisciplinary collaborative models.

### 3.1 Refinement of diagnostic criteria and genotype-phenotype correlation

Traditional diagnostic criteria for PJS underscore the importance of combining clinical features (i.e., mucocutaneous pigmentation and gastrointestinal hamartomatous polyps) with a positive family history. Nevertheless, approximately 10–20% of PJS patients lack STK11 gene mutations and typically present with milder clinical phenotypes. Studies have indicated that, compared to mutation-positive patients, those without STK11 mutations experience a median delay of 5.5 years in the age at first treatment (18.5 years vs. 13.0 years), along with a 47% reduction in the incidence of intussusception and a 30% decrease in polyp burden (10). This observation suggests that, for suspected PJS cases without STK11 mutations, a multigene testing panel (encompassing genes such as PTEN, BRCA1/2, among others) should be employed to rule out other hereditary syndromes and thereby prevent overdiagnosis (11). Meanwhile, personalized monitoring and treatment plans should be developed based on the STK11 gene mutation status.

The association between STK11 genotype and the clinical phenotypes of PJS remains incompletely understood, as this relationship is likely complex and influenced by multiple factors. Research conducted by Jelsig et al. (12) has demonstrated that pathogenic missense variants may influence the age at which polyps initially manifest. Moreover, various types of STK11 gene variations, including single nucleotide variations and copy number variations, exhibit relatively high prevalence and detection rates among PJS patients. Mosaicism is present in approximately 5% of PJS cases, and patients with mosaicism typically exhibit milder phenotypes. These findings collectively suggest that numerous unknown aspects regarding genotype–phenotype correlations in PJS still require further investigation.

### 3.2 Immunology and microbiology

The STK11 gene regulates the AMPK pathway, thereby downregulating the mTOR signaling pathway and serving as a potential therapeutic target for PJS (12). However, this regulatory mechanism is disrupted in PJS patients, and mTOR inhibitors may suppress abnormal pathway activation. The inhibition of polyp growth by agents such as the mTOR inhibitor rapamycin and its derivatives, the COX-2 inhibitor celecoxib, and biguanides represents another ongoing research focus (13). Nevertheless, due to the absence of clinical trials, the efficacy and adverse effects of these drugs remain undetermined, and consequently, they have not yet been clinically utilized at this stage. Therefore, further investigation into the mechanisms underlying PJS development and its related signaling pathways is still essential to identify appropriate therapeutic targets for this disease.

The study by Liu et al. (14) revealed that PJS polyps exhibit an immune microenvironment akin to that of cancerous tissues, characterized by elevated expression of the immune checkpoint gene CD80 and an accumulation of myeloid-derived suppressor cells (MDSCs). These findings suggest that PJS polyps may respond to immunotherapy, with CD80 and MDSCs identified as promising therapeutic targets, thereby paving the way for potential immunotherapeutic applications in PJS patients.

PJS patients exhibit distinct alterations in their gut microbiota and mucosal ecosystem compared to healthy individuals, which are closely associated with disease pathogenesis and clinical manifestations. Mucosal microbiota in PJS patients show reduced diversity and a composition significantly different from fecal microbiota, with notable increases in potentially pathogenic genera such as *Escherichia-Shigella* and *Klebsiella*, alongside decreases in beneficial bacteria (15). These microbial shifts may contribute to disease progression through inflammatory responses and pro-carcinogenic mechanisms. Metabolomic analyses of mucosal samples reveal significant differences in lipid and amino acid metabolism pathways, potentially linked to tumorigenesis. In the gut environment, PJS patients demonstrate greater *β*-diversity changes, with an increased abundance of *Escherichia coli* and decreased *Faecalibacterium prausnitzii* (16). This dysbiosis is more pronounced in patients with intussusception, who exhibit further reductions in *Faecalibacterium prausnitzii* and significant enrichment of propionate metabolism pathways, correlating with disease severity. Additionally, PJS patients show reduced synthesis of short-chain fatty acids (SCFAs), particularly acetate, propionate, and butyrate, which are inversely associated with clinical features such as polyp count and surgical frequency (17). These findings collectively underscore the potential role of gut microbiota and their metabolic functions in PJS pathogenesis.

### 3.3 Innovation in endoscopic techniques and optimization of surgical strategies

For multiple gastrointestinal polyps, endoscopic resection is the primary treatment modality. However, in cases complicated by acute mechanical intestinal obstruction or intussusception, emergency surgical intervention is often necessary. Nevertheless, surgical procedures increase the risk of intra-abdominal infection and may result in short bowel syndrome and intestinal adhesions due to repeated intestinal resections, thereby significantly impairing the patient’s quality of life (18). With advancements in endoscopic technology, small bowel enteroscopy currently represents the optimal approach for detecting and treating small intestinal lesions.

Endoscopic ischemic polypectomy, utilizing clips or a detachable snare, represents the preferred approach for managing pedunculated polyps due to its association with fewer adverse events in comparison to traditional polypectomy techniques (19). Despite the recurrent growth of polyps in patients with PJS, repeated endoscopic resections have been shown to effectively reduce both the total count and average size of polyps over the long term (20). The study by Elfeky et al. (21) demonstrated a significant reduction in the surgical rate from 75 to 8% among patients after polyp resection. Implementing a ‘clean sweep’ strategy during endoscopy, wherein all polyps are removed intraoperatively from the entire intestine, can significantly diminish the necessity for surgical intervention (18). Polyps measuring less than 3 cm can be directly removed using a snare. When performing endoscopic resection on isolated large polyps (≥3 cm), it is essential to assess the polyp’s stalk (22). The thicker the stalk of the polyp, the higher the risk of perforation and bleeding. Epinephrine can be injected into the base of the polyp prior to resection, followed by a piecemeal resection technique. This method enables adequate elevation of the mucosa at the base of the polyp, facilitating surgical removal, and also constricts blood vessels to prevent intraoperative and postoperative hemorrhage. In cases where there are multiple large polyps, a combined surgical procedure incorporating intraoperative colonoscopy can be carried out.

In cases of small-bowel intussusception resulting from PJS polyps, successful endoscopic reduction via double-balloon enteroscopy (DBE) can be accomplished, thus obviating the need for surgical intervention (19). Similarly, when PJS polyps are located in highly mobile intestinal segments, such as the transverse colon or sigmoid colon—particularly when the polyp diameter exceeds 30 mm—endoscopic polypectomy can obviate the need for emergency surgery due to colonic intussusception (23).

Surgical intervention should adopt a conservative strategy as much as possible, such as enterotomy for polypectomy, rather than extensive bowel resection (13). This approach helps to minimize the extent of bowel resection and preserve adequate intestinal function. Prior to surgery, detailed assessment of polyp distribution, size, and morphology can be conducted using endoscopic techniques such as VCE and DBE, providing crucial information for surgical planning. This aids in determining the surgical scope and avoiding unnecessary bowel resection. During the surgical procedure, intraoperative endoscopy can be employed to observe the internal condition of the intestines in real-time, ensuring complete polyp resection (24). Particularly when dealing with multiple polyps or deep-seated polyps, endoscopic assistance significantly enhances the precision and safety of the surgery.

### 3.4 Surgical risk scoring system for intussusception

Xiao et al. (25) developed the first surgical risk scoring system for PJS based on three objective indicators: abdominal pain severity (none/mild vs. moderate/severe), polyp diameter (<4 cm vs. 4–6 cm vs. >6 cm), and intussusception length (<8 cm vs. 8–16 cm vs. >16 cm). Each risk factor was assigned varying weights (0–9 points), stratifying patients into three tiers: low-risk (0–3 points), moderate-risk (4–6 points), and high-risk (7–9 points). For low-risk patients, endoscopic treatment is recommended as the first-line option due to its high success rate (95.56%), which helps avoid unnecessary surgery; moderate-risk patients require treatment decisions that integrate endoscopist experience and patient preferences, allowing for attempted endoscopic therapy but necessitating a backup surgical plan; high-risk patients should be prioritized for surgery given the high failure rate of endoscopic treatment (83.33%) and the potential for delayed definitive care. This system facilitates preoperative risk stratification to guide tailored therapeutic strategies for enteroenteric intussusception in PJS.

### 3.5 Malignant transformation and surveillance

Patients with PJS exhibit tumor susceptibility, and the risk of malignant transformation escalates with age, reaching as high as 85% by 70 years of age (26). The organs with a high risk of malignant transformation include the gastrointestinal tract (39% for the colorectal region, 29% for the stomach, and 13% for the small intestine), breast (24–54%), pancreas (11–36%), ovaries (21%), uterus (9%), testes (9%), and lungs (7–17%) (13). Cancer risk increases significantly starting at age 40, necessitating lifelong surveillance.

Clinical guidelines propose management strategies for PJS tailored to age-specific and organ-specific malignancy risks (13). Pediatric monitoring recommends high-quality colonoscopy and gastroscopy every 2–3 years, with follow-up intervals shortened to 1–2 years if polyps are detected. Routine screening is resumed after age 18 if no polyps are present. For small intestine surveillance, VCE or CT/MRI enterography (MRE) is recommended every 2–3 years, with interval adjustments based on polyp characteristics (size, number, and pathology). Females should undergo annual pelvic ultrasound and Pap smears starting at age 8, while males require annual testicular examinations to monitor for precocious puberty or tumor signs.

Adult surveillance includes repeat colonoscopy, gastroscopy, and small bowel VCE/MRE every 2–3 years. Annual pancreatic EUS or MRI/MRCP screening is initiated at ages 30–35 (with shortened intervals for high-risk individuals), alongside annual breast MRI and mammography starting at age 30. Smoking cessation education is provided, as no specific lung screening is recommended. After age 50, enhanced screening for pancreatic MRI, breast MRI, and lung CT is advised, with frequency tailored to family history or high-risk features.

### 3.6 Lessons from the case

In this case, the patient’s oral pigmentation—a 1.5 mm black macule on the lower lip—was overlooked preoperatively due to its subtle presentation and insufficient clinical awareness of its diagnostic significance. This oversight contributed to a delayed diagnosis of PJS-related intussusception, as clinicians initially failed to associate the recurrent abdominal pain with an underlying hereditary polyposis syndrome. During hospitalization, conservative management was prioritized despite radiographic evidence of intussusception. Early endoscopic reduction via DBE was not performed, necessitating eventual emergency open surgery. For hemodynamically stable patients with small-bowel intussusception, timely endoscopic intervention—which has demonstrated a high success rate (>95% in low-risk cases)—could have obviated surgical intervention. Furthermore, preoperative STK11 mutation testing was not conducted, limiting opportunities for genotype–phenotype risk stratification.

## 4 Conclusion

PJS poses significant diagnostic and management challenges due to its rarity and associated malignancy risks. Early identification and intervention are crucial to prevent severe complications such as intussusception and malignancies. This case report and literature review underscore the importance of a multidisciplinary approach in PJS management, integrating genetic testing, endoscopic expertise, and surgical strategies. Personalized treatment plans based on genotype–phenotype correlations and risk stratification are advocated. Future research should focus on elucidating unknown aspects of genotype–phenotype relationships, identifying novel therapeutic targets, and refining endoscopic techniques to enhance patient care and reduce malignancy incidence. Lifelong surveillance remains paramount to ensure early detection and intervention, ultimately improving the quality of life and prognosis for PJS patients.

## Data Availability

The original contributions presented in the study are included in the article/supplementary material, further inquiries can be directed to the corresponding authors.
